# Facial stimulation induces long-term depression at cerebellar molecular layer interneuron–Purkinje cell synapses *in vivo* in mice

**DOI:** 10.3389/fncel.2015.00214

**Published:** 2015-06-09

**Authors:** Yan-Hua Bing, Mao-Cheng Wu, Chun-Ping Chu, De-Lai Qiu

**Affiliations:** ^1^Cellular Function Research Center, Yanbian UniversityYanji, Jilin Province, China; ^2^Department of Physiology and Pathophysiology, College of Medicine, Yanbian UniversityYanji, Jilin Province, China; ^3^Department of Osteology, Affiliated Hospital of Yanbian UniversityYanji, Jilin Province, China

**Keywords:** cerebellar Purkinje cell, molecular layer interneuron, sensory stimulation, plasticity, NMDA receptor, endocannabinoids receptor, *in vivo* cell-attached recording

## Abstract

Cerebellar long-term synaptic plasticity has been proposed to provide a cellular mechanism for motor learning. Numerous studies have demonstrated the induction and mechanisms of synaptic plasticity at parallel fiber–Purkinje cell (PF–PC), parallel fiber–molecular layer interneurons (PF–MLI) and mossy fiber–granule cell (MF–GC) synapses, but no study has investigated sensory stimulation-evoked synaptic plasticity at MLI–PC synapses in the cerebellar cortex of living animals. We studied the expression and mechanism of MLI–PC GABAergic synaptic plasticity induced by a train of facial stimulation in urethane-anesthetized mice by cell-attached recordings and pharmacological methods. We found that 1 Hz, but not a 2 Hz or 4 Hz, facial stimulation induced a long-term depression (LTD) of GABAergic transmission at MLI–PC synapses, which was accompanied with a decrease in the stimulation-evoked pause of spike firing in PCs, but did not induce a significant change in the properties of the sensory-evoked spike events of MLIs. The MLI–PC GABAergic LTD could be prevented by blocking cannabinoid type 1 (CB1) receptors, and could be pharmacologically induced by a CB1 receptor agonist. Additionally, 1 Hz facial stimulation delivered in the presence of a metabotropic glutamate receptor 1 (mGluR1) antagonist, JNJ16259685, still induced the MLI–PC GABAergic LTD, whereas blocking N-methyl-D-aspartate (NMDA) receptors during 1 Hz facial stimulation abolished the expression of MLI–PC GABAergic LTD. These results indicate that sensory stimulation can induce an endocannabinoid (eCB)-dependent LTD of GABAergic transmission at MLI–PC synapses via activation of NMDA receptors in cerebellar cortical Crus II *in vivo* in mice. Our results suggest that the sensory stimulation-evoked MLI–PC GABAergic synaptic plasticity may play a critical role in motor learning in animals.

## Introduction

Synaptic plasticity is a modification of synaptic strength, which is important to the formation and stability of neuronal circuits. Cerebellar long-term synaptic plasticity can be induced at parallel fiber–Purkinje cell (PF–PC), parallel fiber–molecular layer interneuron (PF–MLI), mossy fiber–granule cell (MF–GC) and MLI–PC synapses under *in vitro* conditions, and has been proposed as a cellular mechanism for motor learning (Grasselli and Hansel, 2014).

The cerebellar cortex includes the molecular layer (ML), Purkinje cell layer (PCL) and granule cell layer (GCL), which are mainly populated by molecular interneurons (MLI), Purkinje cells (PC), and granule cells (GC) and Golgi cells, respectively (Eccles et al., 1967). Synaptic plasticity at PF–PC, PF–MLI, and MF–GC synapses has been widely studied in cerebellar slices (Ito and Kano, 1982; Qiu and Knöpfel, 2007, 2009; D’Errico et al., 2009; Piochon et al., 2010; Garrido et al., 2013; D’Angelo, 2014; van Beugen et al., 2014; Yamazaki et al., 2015) and in living animals (Roggeri et al., 2008; Márquez-Ruiz and Cheron, 2012; Chu et al., 2014). At MLI–PC synapses, three types of plasticity have been induced by postsynaptic depolarization under *in vitro* conditions; depolarization-induced potentiation of inhibition (DPI), depolarization-induced suppression of inhibition (DSI) and rebound potentiation (RP; Hirano, 2013). DSI is a type of short-lasting suppression in presynaptic GABA release mediated by endocannabinoids (eCB), which are released from a PC and bind to presynaptic CB1 receptors (Llano et al., 1991a; Yoshida et al., 2002). In contrast, DPI is a long-term potentiation (LTP) of presynaptic GABA release mediated by glutamate release from a postsynaptic PC, which then binds to presynaptic NMDA receptors (Duguid and Smart, 2004). RP occurs postsynaptically and lasts longer; it is induced by a Ca^2+^-dependent upregulation of GABA_A_ receptor activity on PCs (Kano et al., 1992, 1996; Kawaguchi and Hirano, 2000, 2007; Tanaka et al., 2013; Hirano and Kawaguchi, 2014). Moreover, repetitive stimulation of CFs can induce GABAergic transmission LTP in PCs (Kano et al., 1996; Kawaguchi and Hirano, 2002). The GABAergic transmission LTP at MLI–PC synapses requires an enhanced postsynaptic Ca^2+^ transient in PCs through voltage-gated Ca^2+^ channels and inositol 1, 4, 5-triphophate (IP3)-mediated Ca^2+^ release from internal stores. The increased Ca^2+^ transient activates calmodulin-dependent protein kinase II (CaMKII), which in turn regulates GABAergic transmission at MLI–PC synapses via GABA_A_ receptors (Kano et al., 1992, 1996; Kawaguchi and Hirano, 2007).

Similar to the synaptic plasticity at PF–PC, PF–MLI, and MF–GC synapses, MLI–PC synaptic plasticity may also be related to cerebellar motor learning (Hirano and Kawaguchi, 2014). PC-specific deletion of GABA_A_ receptor γ2 subunits, which removes all inhibition of PCs, affects both phase reversal learning, and gain and phase consolidation of the vestibulo-ocular reflex (Wulff et al., 2009; Seja et al., 2012). Moreover, CaMKII deletion in PCs, may also affect the MLI–PC synapse indirectly and influence both gain increase and decrease in vestibulo-ocular reflex learning (Hansel et al., 2006; Schonewille et al., 2010). Therefore, MLI–PC synaptic plasticity might be related to the coding of cerebellar cortical information and motor learning.

Collectively, MLI–PC synaptic plasticity has been studied in cerebellar slices, but the mechanisms of sensory stimulation-evoked synaptic plasticity at MLI–PC synapses in the cerebellar cortex of living animals are currently unknown. Therefore, we studied the mechanism of MLI–PC GABAergic synaptic plasticity induced by a train of facial stimulation in urethane-anesthetized mice by physiological and pharmacological methods. Our results showed that 1 Hz, but not 2 Hz or 4 Hz, facial stimulation induced GABAergic transmission LTD at MLI–PC synapses, accompanied with a decrease in the sensory-evoked pause of spike firing. The MLI–PC GABAergic LTD could be prevented by blocking CB1 receptors, and could be pharmacologically induced by a CB1 receptor agonist. MLI–PC GABAergic LTD was not blocked by an mGluR1 antagonist, but was abolished by blockade of NMDA receptors during 1 Hz facial stimulation. These results indicate that sensory stimulation induces an eCB-dependent LTD of GABAergic transmission at MLI–PC synapses via activation of NMDA receptors *in vivo* in mice.

## Materials and Methods

### Anesthesia and Surgical Procedures

The anesthesia and surgical procedures have been described previously (Chu et al., 2011a,b). The experimental procedures were approved by the Animal Care and Use Committee of Jilin University and were in accordance with the animal welfare guidelines of the U.S. National Institutes of Health. The permit number is SYXK (Ji) 2007-0011. Adult (6–8-week-old) HA/ICR mice were anesthetized with urethane (1.3 g/kg body weight, i.p.). Mice were tracheotomized to avoid respiratory obstruction. On a custom-made stereotaxic frame, soft tissue was retracted to gain access to the dorsal portion of the occipital bone. A watertight chamber was created and a 1–1.5-mm craniotomy was drilled to expose the cerebellar surface corresponding to Crus II. The brain surface was constantly superfused with oxygenated artificial cerebrospinal fluid (ACSF: 125 mM NaCl, 3 mM KCl, 1 mM MgSO_4_, 2 mM CaCl_2_, 1 mM NaH_2_PO_4_, 25 mM NaHCO_3_, and 10 mM D-glucose) with a peristaltic pump (Gilson Minipulse 3; Villiers, Le Bel, France) at 0.4 ml/min. Rectal temperature was monitored and maintained at 37.0 ± 0.2°C using body temperature equipment.

### Cell-Attached Recording and Facial Stimulation

Cell-attached recordings from PCs were performed with an Axopatch-200B amplifier (Molecular Devices, Foster City, CA, USA). The signals of PC cell-attached recordings were acquired through a Digidata 1440 series analog-to-digital interface on a personal computer using Clampex 10.3 software (Molecular Devices). Patch pipettes were made with a puller (PB-10; Narishige, Tokyo, Japan) from thick-wall borosilicate glass (GD-1.5; Narishige). Recording electrodes were filled with ACSF, with resistances of 3–5 MΩ. The cell-attached recordings from PCs were performed at depths of 150–200 μm under the pia mater membrane, and were identified by regular spontaneous simple spikes (SSs) accompanied with irregular complex spikes. The MLIs were roughly identified by irregularly spontaneous spike activity and the depth of the recording site under the cell-attached recording condition (Chu et al., 2012).

**Figure 1:**
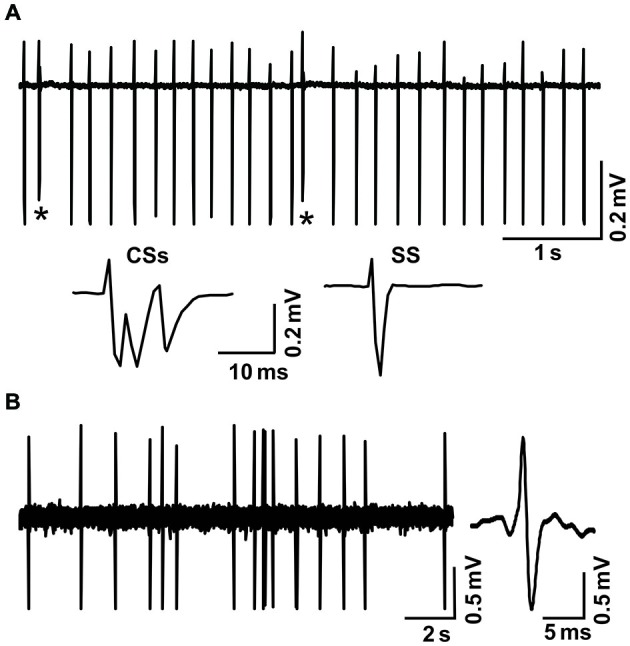
**Cell-attached recordings showing the characterization of cerebellar cortical PCs and MLIs *in vivo* in mice. (A)** Upper, representative cell-attached recording traces showing the spontaneous activity of a cerebellar PC. Lower, enlarged traces of CSs and a simple spike (SS). CSs are indicated by*. **(B)** Left, representative cell-attached recording traces showing the spontaneous activity of a cerebellar MLI. Right, enlarged traces of spike firing.

Facial stimulation was performed by air-puff (10 ms, 60 psi) of the ipsilateral whisker pad through a 12-gauge stainless steel tube connected with a pressurized injection system (Picospritzer® III; Parker Hannifin Co., Pine Brook, NJ, USA). The air-puff stimulations were controlled by a personal computer, and were synchronized with the electrophysiological recordings and delivered at 0.05 Hz via a Master 8 controller (A.M.P.I., Jerusalem, Israel) and Clampex 10.3 software. The facial stimulation-evoked MLI–PC synaptic response has been demonstrated in our previous studies (Chu et al., 2011a,b); the response is a sequence of negative components (N1) followed by a positive component (P1) accompanied with a pause of SS firing (Figure 1A). For the induction of MLI–PC synaptic plasticity, 240 pulses of air-puff stimulation (10 ms, 60 psi) were delivered at 1 Hz, 2 Hz, and 4 Hz. The induction stimulation was delivered 10 min after the recording became stable.

### Chemicals

The reagents used were D-aminophosphonovaleric acid (D-APV), the NMDA receptor antagonist; N-(piperidin-1-yl)-5-(4-iodophenyl)-1-(2, 4-di-chlorophenyl)-4-methyl-1H-pyrazole-3-carboxamide (AM251), for blocking endocannabinoid CB1 receptors; (3, 4-dihydro-2H-pyrano [2, 3-b]quinolin-7-yl)-(cis-4-methoxycyclohexyl)-methanone [JNJ16259685 (JNJ)], a group 1 metabotropic glutamate receptor antagonist; and (R)-(+)-[2, 3-dihydro-5-methyl-3-(4-morpholinylmethyl) pyrrolo[1, 2, 3-de]-1, 4-benzoxazin-6-yl]-1-naphthalenylmethanone mesylate (WIN55212-2), a CB1 receptor agonist. All chemicals were purchased from Sigma-Aldrich (Shanghai, China). The drugs were dissolved in ACSF, and applied directly onto the cerebellar surface by a peristaltic pump (0.5 ml/min).

### Data Analysis

The electrophysiological data were analyzed using Clampfit 10.3 software (Molecular Devices, Foster City, CA, USA). Values are expressed as the mean ± SEM. ANOVA (posthoc multiple comparison; SPSS software) was used to determine the level of statistical significance among groups of data. *P*-values below 0.05 were considered statistically significant.

## Results

### Facial Stimulation (1 Hz) Induces GABAergic Transmission LTD at PCs in the Mouse Cerebellar Cortex

**Figure 2:**
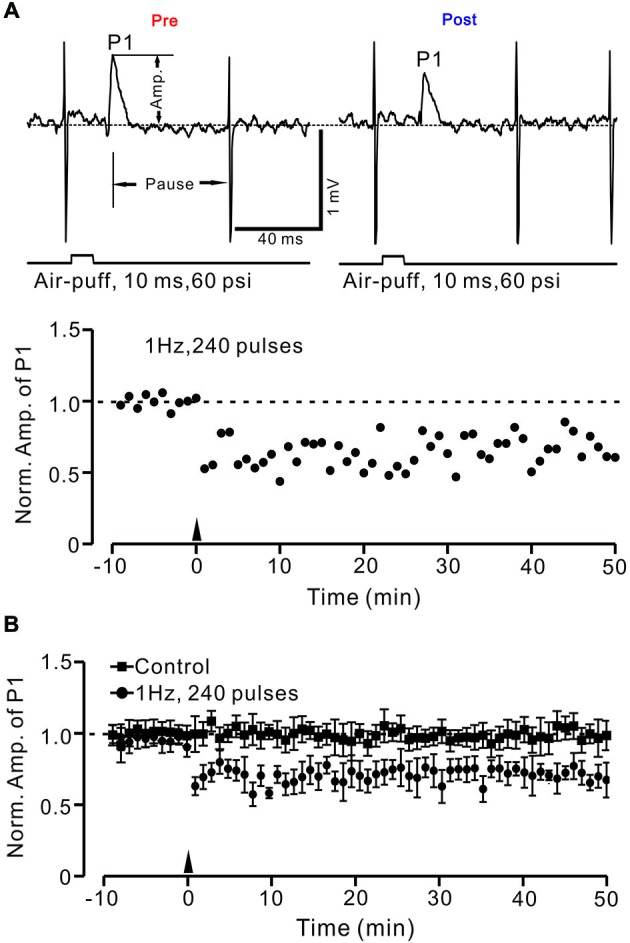
**Facial stimulation (1 Hz) induces long-term depression (LTD) of GABAergic transmission at PCs in mouse cerebellar cortex. (A)** Upper, representative cell-attached recording traces showing air-puff stimulation (10 ms, 60 psi)-evoked responses in a cerebellar PC before (pre) and after (post) delivering 1 Hz (240 pulses) stimulation. Note that both amplitude of the positive component (P1) and the pause of SS firing are significantly decreased after 1 Hz stimulation. The lower panel shows the time course of normalized amplitude of P1 (shown in upper) before and after delivery of 1 Hz facial stimulation (arrow head). **(B)** Summary of normalized P1 amplitude under control conditions (squares; *n* = 7) and delivery of 1 Hz facial stimulation (arrow head; circles; *n* = 8). Note that air-puff stimulation at 1 Hz induced LTD of P1 in cerebellar PCs. Data points are mean ± SEM.

Under cell-attached recording conditions, PCs were identified by the presence of regular spontaneous SS firing activity accompanied with complex spikes. These PCs responded to air-puff stimulation (10 ms; 60 psi), which was expressed as a sequence of a negative component (N1) followed by a positive component (P1), accompanied by a pause in SS firing (Figure 2A). According to our previous studies (Chu et al., 2011a), N1 is a component of the PF volley, whereas P1 is sensitive to GABA_A_ receptor antagonist, which identifies it as MLI–PC GABAergic synaptic transmission onto the PC. Based on the frequency properties of PCs (Bing et al., 2015b), we first examined whether MLI–PC GABAergic synaptic plasticity could be evoked by 1 Hz facial stimulation (240 pulses). This repetitive stimulation produced a persistent depression of MLI–PC GABAergic synaptic transmission, which was expressed as a decrease in P1 amplitude for over 50 min (Figure 2A). The normalized amplitude of P1 was decreased to 73.6 ± 12.4% of baseline for 40–50 min after 1 Hz facial stimulation (*P* < 0.05, *n* = 7, Figure 1B). In contrast, the normalized amplitude of P1 was 102.2 ± 10.3% of baseline at 40–50 min under control conditions (*P* > 0.05, *n* = 8, Figure 2B). Additionally, the normalized amplitude of N1 at 40–50 min after 1 Hz facial stimulation was 99.6 ± 25.3% of baseline, which did not change significantly after the repetitive stimulation (*P* > 0.05, *n* = 8, data not shown). These results indicated that 1 Hz facial stimulation induced GABAergic transmission LTD at PCs in the mouse cerebellar cortex.

**Figure 3:**
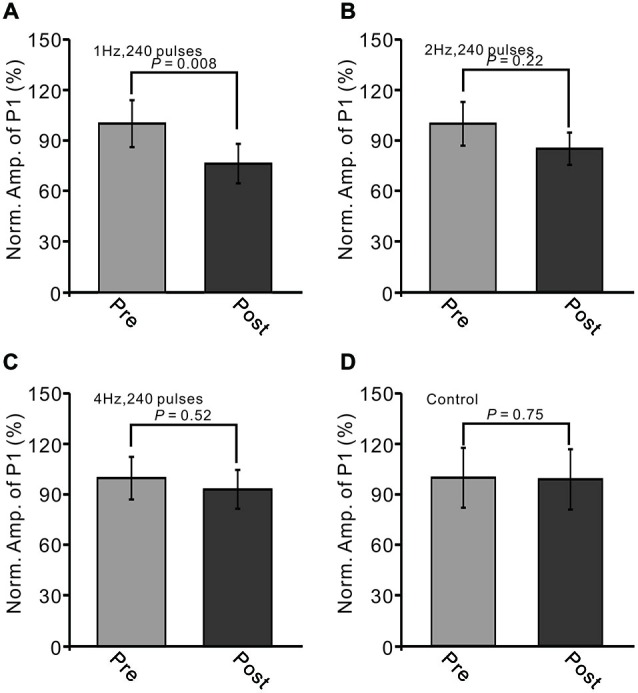
**LTD of sensory-evoked P1 at PCs was induced by 1 Hz, but not by 2 Hz or 4 Hz. (A)** Bar graph showing normalized amplitude of P1 before (pre) and after (post) delivery of 2 Hz (240 pulses) stimulation. **(B)** Summary of data showing normalized amplitude of P1 before (pre) and after (post) 2 Hz (240 pulses) stimulation. **(C)** Pooled data showing normalized amplitude of P1 before (pre) and after (post) 4 Hz (240 pulses) stimulation. **(D)** Bar graph showing normalized amplitude of P1 under control conditions (without train of facial stimulation). Note that the amplitude of P1 was depressed after 1 Hz stimulation, but not after 2 or 4 Hz stimulation.

Next, we examined whether the LTD of GABAergic transmission at PCs could be induced by 2 Hz and 4 Hz facial stimulation. As shown in Figure 3, the normalized amplitude of P1 at 40–50 min after 1 Hz facial stimulation was decreased to 71.5 ± 8.9% of baseline (100.0 ± 11.6%; *P* < 0.05, *n* = 7; Figure 3A). However, after 2 Hz stimulation (*P* > 0.05, *n* = 7 in each group, Figure 3B), the normalized amplitude of P1 at 40–50 min was 84.8 ± 9.5% of baseline (99.6 ± 12.9%). After 4 Hz facial stimulation (*P* > 0.05, *n* = 7 in each group, Figure 3C), the normalized amplitude of P1 at 40–50 min was 93.3 ± 11.6% of baseline (100.0 ± 12.7%), which was similar to that under control conditions (99.9 ± 18.6% of baseline; *P* > 0.05, *n* = 7).

**Figure 4:**
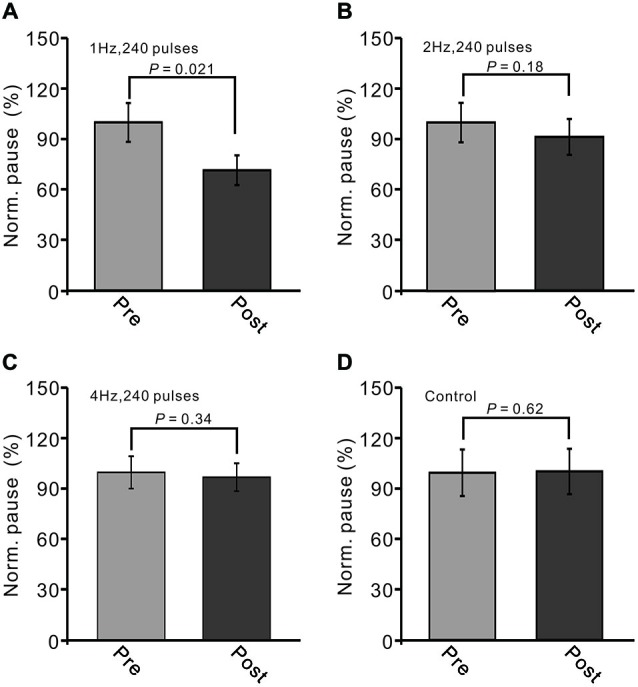
**LTD of sensory-evoked P1 at PCs was accompanied with a decrease in the pause of SS firing. (A)** Bar graph showing normalized pause of SS firing before (pre) and after (post) delivery of 1 Hz (240 pulses) stimulation.** (B)** Pooled data showing normalized pause of SS firing before (pre) and after (post) delivery of 2 Hz (240 pulses) stimulation. **(C)** Summary of data showing normalized values of the pause before (pre) and after (post) delivering 4 Hz (240 pulses) stimulation. **(D)** Bar graph showing normalized pause firing values under control conditions (without train of facial stimulation). Note that the amplitude of the pause of SS firing was decreased after 1 Hz stimulation, but not after 2 Hz or 4 Hz stimulation.

Additionally, we evaluated the change in the stimulation-evoked pause of spike firing after the trains of facial stimulation. The normalized value of the pause of spike firing at 40–50 min after 1 Hz facial stimulation was decreased to 76.3 ± 11.7% of baseline (100.0 ± 13.9%; *P* < 0.05, *n* = 7; Figure 4A). As with the normalized amplitude of P1, the normalized pause of spike firing at 40–50 min after 2 Hz facial stimulation was 91.4 ± 13.0% of baseline (99.8 ± 13.4%; *P* > 0.05, *n* = 7 in each group, Figure 4B), and the normalized pause of spike firing over 40–50 min after 4 Hz facial stimulation was 97.2 ± 8.3% of baseline (100.0 ± 9.6%; *P* > 0.05, *n* = 7 in each group, Figure 4C), which was similar to the normalized pause of spike firing over 40–50 min under control conditions (99.9 ± 18.6% of baseline; *P* > 0.05, *n* = 7; Figure 4D). These results indicated that LTD of GABAergic transmission at MLI–PC synapses could be induced by sensory stimulation at 1 Hz, but not at 2 Hz or 4 Hz.

### Facial Stimulation (1 Hz) Induced LTD of GABAergic Transmission at PCs, but Without Significant Change in the Properties of Sensory-Evoked Spike Events in MLIs

**Figure 5:**
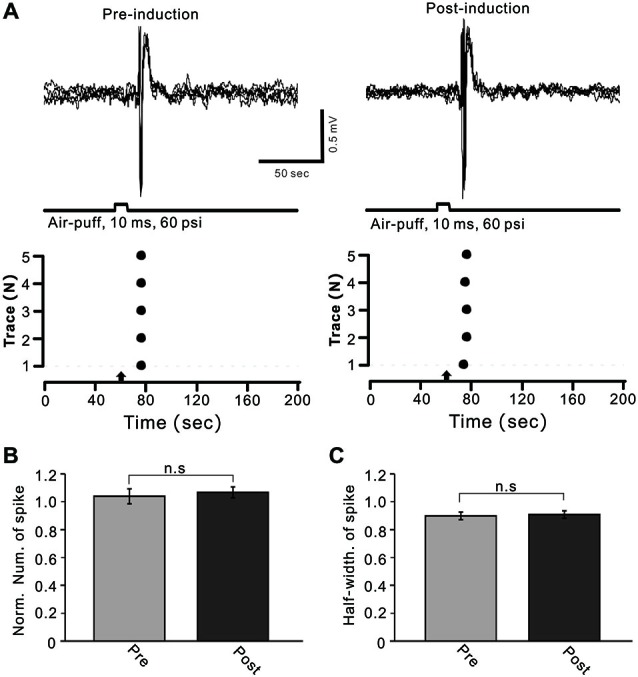
**Facial stimulation had a lesser effect on cerebellar MLI activity. (A)** Upper, superposition of five representative recording epochs showing air-puff stimulation (10 ms, 60 psi)-evoked spike firing in an MLI before (left) and after (post) delivery of 1 Hz (240 pulses) stimulation. The lower panel shows the master plot of spike events derived from traces of the upper panel. **(B)** Summary of normalized number of spike events before (Pre, red) and after delivery of 1 Hz stimulation (Post; *n* = 6). **(C)** Pooled data showing the normalized half-width of the sensory-evoked spike events before (Pre) and after delivering 1 Hz stimulation (Post; *n* = 6). Note that air-puff stimulation at 1 Hz did not induce a significant change in the properties of the sensory-evoked spike events on MLIs.

MLIs receive sensory information from the MF–GC–PF pathway, and LTD of MLI–PC GABAergic transmission can be induced by the change of sensory stimulation-evoked MLI responses. Therefore, we examined the effects of 1 Hz facial stimulation on the activities of cerebellar MLIs. As with our previous study (Chu et al., 2012), cerebellar MLIs were identified by irregular spike firing and depth of recording sites (Figure 1B). Under cell-attached recording conditions, cerebellar MLIs exhibited spike firing in response to air-puff stimulation of the ipsilateral whisker pad (10 ms; 60 m psi; Figure 5A). After 1 Hz facial stimulation, the normalized number of evoked spike events at 40–50 min was 105.7 ± 5.3% of baseline (100.9 ± 3.9%), which was not significantly different from baseline (*P* > 0.05, *n* = 7 in each group, Figures 5A,B). Moreover, the mean half-width of the evoked spike events at 40–50 min was 0.90 ± 0.027 ms after 1 Hz facial stimulation, which was not significantly different from baseline (0.91 ± 0.026 ms; *P* > 0.05, *n* = 7 in each group, Figures 5A,C). These results indicated that air-puff stimulation at 1 Hz did not induce significant change in the properties of the sensory-evoked spike events on MLIs, suggesting that LTD of GABAergic transmission was induced by the train of sensory stimulation at MLI–PC synapses.

### Induction of MLI–PC GABAergic LTD Requires Activation of CB1 Receptors

**Figure 6:**
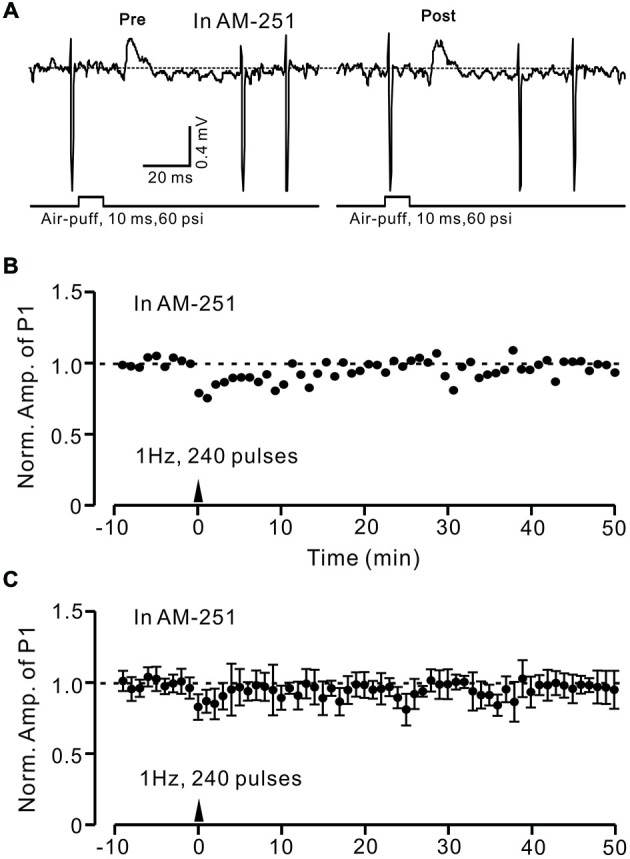
**Facial stimulation-induced LTD of GABAergic transmission at PCs was abolished by blocking cannabinoid type 1 (CB1) receptor activity. (A)** Representative cell-attached recording traces showing air-puff stimulation (10 ms, 60 psi)-evoked responses in a cerebellar PC before (pre) and after (post) delivering 1 Hz (240 pulses) stimulation delivered in the presence of CB1 antagonist, AM251 (5 μM). **(B)** Time course of normalized amplitude of P1 (shown in **A**) before and after delivery of 1 Hz facial stimulation (arrow head) delivered in the presence of AM251. **(C)** Summary of normalized amplitude of P1 before and after delivery of 1 Hz (240 pulses) stimulation (arrow head; *n* = 7) delivered in the presence of AM251. Note that air-puff stimulation (1 Hz)-induced LTD of P1 was blocked by application of a CB1 receptor antagonist. Data points are mean ± SEM.

**Figure 7:**
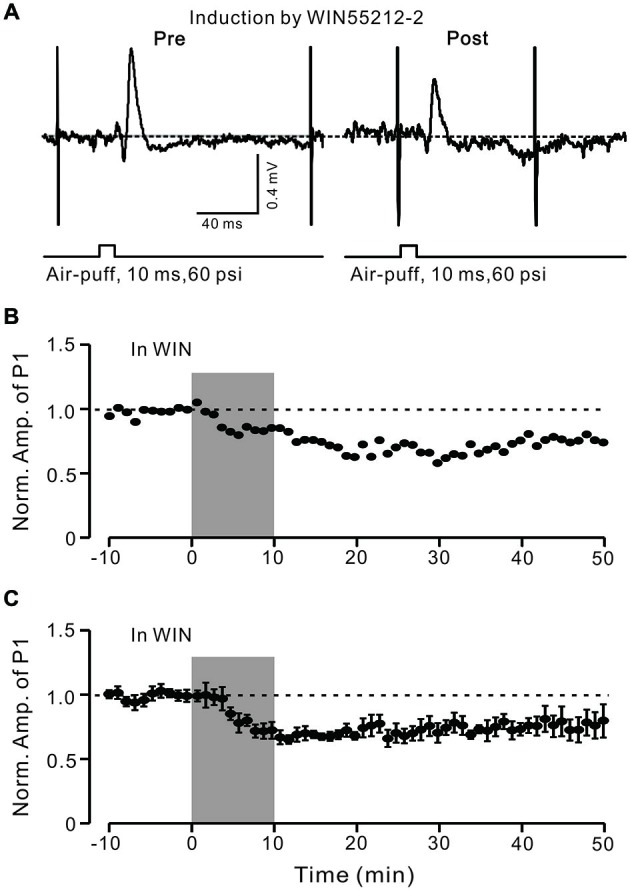
**LTD of GABAergic transmission at PCs was induced by a CB1 receptor agonist. (A)** Representative cell-attached recording traces showing air-puff stimulation (10 ms, 60 psi)-evoked responses in a cerebellar PC before (left) and after application of CB1 agonist WIN55212-2 (5 μM; 10 min). **(B)** Time course of normalized amplitude of P1 (shown in **A**) before and after application of CB1 agonist WIN55212-2 (5 μM; 10 min; shadow). **(C)** Pooled data showing the average normalized amplitude of P1 before and after application of CB1 agonist WIN55212-2 (5 μM; 10 min; shadow; *n* = 7). Note that application of CB1 agonist WIN55212-2 for 10 min induced LTD of P1. Data points are mean ± SEM.

In the cerebellar cortex, eCBs are generated and released from PCs and MLIs by trains of PF stimulation via activation of mGluR1 and NMDA receptors (Beierlein and Regehr, 2006; Soler-Llavina and Sabatini, 2006), and are considered to be related to PF–PC presynaptic plasticity (Qiu and Knöpfel, 2009; Chu et al., 2014). Therefore, we examined the effect of the CB1 receptor antagonist on the induction of 1 Hz facial stimulation-induced LTD of GABAergic transmission at PCs. Blockade of endocannabinoid CB1 receptors with AM251 completely prevented the induction of 1 Hz facial stimulation-induced LTD of GABAergic transmission at MLI–PC synapses (Figures 6A,B). In the presence of AM251, the mean amplitude of P1 40–50 min after the trains of 1 Hz facial stimulation were delivered was 95.9 ± 9.4% (*n* = 6) of baseline (100 ± 5.2%; Figures 6A,C; *P* < 0.05), indicating that 1 Hz facial stimulation induced LTD of GABAergic transmission at MLI–PC synapses via CB1 receptor activation. Furthermore, we applied the CB1 receptor agonist, WIN55212-2, to examine whether LTD of GABAergic transmission at MLI–PC synapses could be induced by direct activation of CB1 receptors. As shown in Figure 7, application of WIN55212-2 (5 μM) for 10 min, induced a long-term depression in P1 amplitude (Figures 7A,B); the normalized P1 amplitude was 76.0 ± 5.9% of baseline (100 ± 4.5%) at 40–50 min (*P* < 0.05, *n* = 7; Figure 7C). These results indicated that induction of MLI–PC GABAergic LTD required CB1 receptor activation, which could be achieved by pharmacological activation.

### Induction of MLI–PC GABAergic LTD Requires Activation of NMDA Receptors, but not mGluR1

**Figure 8:**
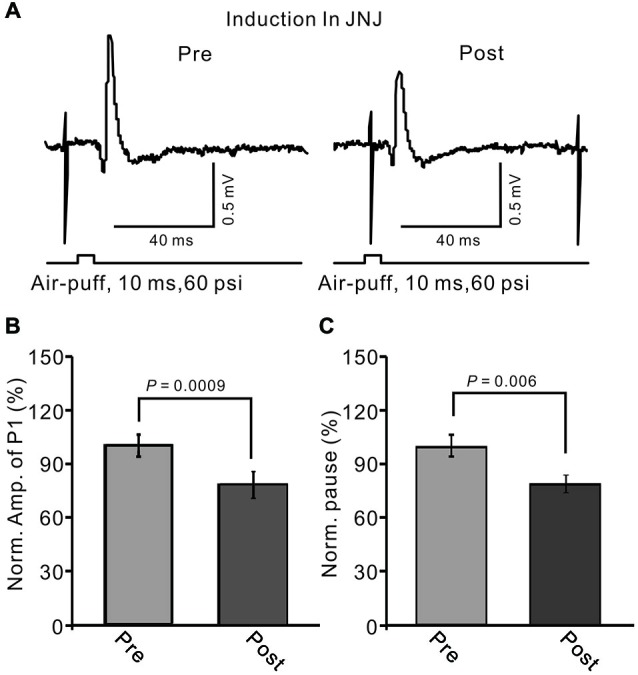
**Blocking metabotropic glutamate receptor 1 (mGluR1) during 1 Hz stimulation failed to prevent the LTD of GABAergic transmission at PCs. (A)** Upper, representative cell-attached recording traces showing air-puff stimulation (10 ms, 60 psi)-evoked responses in a PC before (pre) and after (post) 1 Hz (240 pulses) stimulation delivered in the presence of mGluR1 antagonist, JNJ16259685 (10 μM, right panel). **(B)** Summary of data showing normalized amplitude of P1 before (pre) and after (post) delivery of 1 Hz (240 pulses) stimulation. **(C)** Pooled data showing the normalized pause of SS firing before (pre) and after (post) delivery of 1 Hz (240 pulses) stimulation. Note that the amplitude of P1 was still depressed after 1 Hz stimulation without mGluR1 activity.

In the cerebellar cortex, PCs and MLIs generate and release eCB by mGluR1 activation (Brown et al., 2003; Beierlein and Regehr, 2006; Soler-Llavina and Sabatini, 2006). Therefore, we examined whether MLI–PC synaptic transmission LTD could be induced by 1 Hz stimulation delivered with blocking of mGluR1. When mGluR1 activity was blocked during 1 Hz stimulation, a persistent depression of MLI–PC GABAergic synaptic transmission was still induced (Figure 8A). The normalized amplitude of P1 was decreased to 78.3 ± 7.65% of baseline (100.0 ± 5.9%) at 40–50 min after 1 Hz facial stimulation (*P* < 0.05; *n* = 6; Figure 8B). The normalized value of the pause of spike firing at 40–50 min after 1 Hz facial stimulation was decreased to 81.2 ± 5.1% of baseline (102.2 ± 6.1%; *P* < 0.05, *n* = 6; Figure 8C). These results indicate that mGluR1 activity contributed less to the induction of MLI–PC GABAergic LTD under *in vivo* conditions.

**Figure 9:**
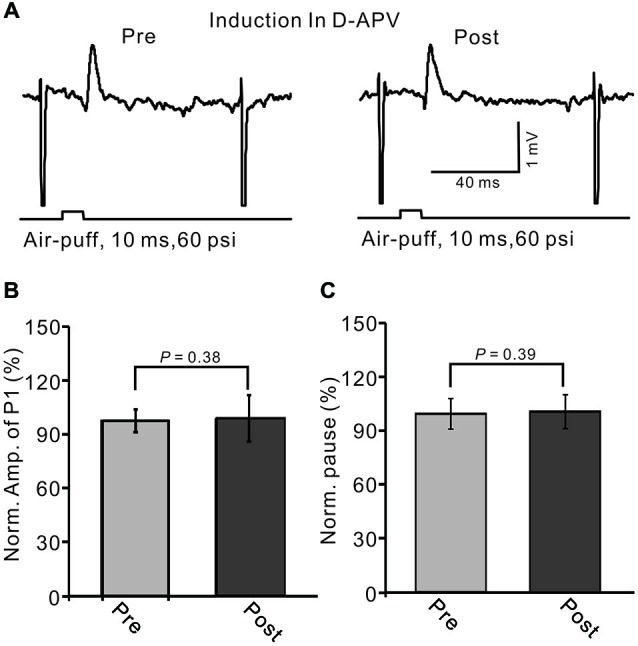
**Blocking N-methyl-D-aspartate (NMDA) receptors during 1 Hz stimulation abolished the LTD of GABAergic transmission at PCs. (A)** Upper, representative cell-attached recording traces showing air-puff stimulation (10 ms, 60 psi)-evoked responses in a PC before (pre) and after (post) delivery of 1 Hz (240 pulses) stimulation delivered in the presence of NMDA receptor antagonist, D-APV (50 μM). **(B)** Summary of data showing normalized amplitude of P1 before (pre) and after (post) delivery of 1 Hz (240 pulses) stimulation. **(C)** Pooled data showing the normalized pause of SS firing before (pre) and after (post) delivery of 1 Hz (240 pulses) stimulation.

NMDA receptor activation is required for eCB-dependent presynaptic LTD induction, both in mouse cerebellar slices (Qiu and Knöpfel, 2009) and in living animals (Chu et al., 2014). Thus, we further examined whether the LTD of MLI–PC synaptic transmission could be induced by 1 Hz stimulation delivered without the activity of NMDA receptors. When NMDA receptor activity was blocked during 1 Hz stimulation with D-APV (50 μM), the LTD of MLI–PC GABAergic synaptic transmission was not induced by facial stimulation (Figure 9A). The normalized amplitude of P1 was 101.3 ± 12.9% of baseline (100.0 ± 6.3%) at 40–50 min after 1 Hz facial stimulation (*P* > 0.05, *n* = 6; Figure 9B). The normalized value of the pause of spike firing at 40–50 min was 102.0 ± 9.3% of baseline (100.0 ± 8.6%) after 1 Hz facial stimulation (*P* > 0.05, *n* = 6; Figure 9C). These results indicate that the induction of MLI–PC GABAergic LTD under *in vivo* conditions occurs via the activation of NMDA receptors.

## Discussion

Our major finding is that 1 Hz, but not 2 Hz or 4 Hz, facial stimulation induces GABAergic transmission LTD at MLI–PC synapses. The MLI–PC GABAergic LTD could be prevented by blocking CB1 receptors, and could be mimicked by pharmacological activation of CB1 receptors. Additionally, the MLI–PC GABAergic LTD was abolished by blocking NMDA receptor activity during inductive stimulation. Our present results indicate that sensory stimulation induces an eCB-dependent LTD of GABAergic transmission at MLI–PC synapses via NMDA receptor activation in living animals.

### Repeated Facial Stimulation Induces LTD of GABAergic Transmission at MLI–PC Synapses *In Vivo* in Mice

MLI–PC synaptic plasticity induced by postsynaptic depolarization at SC–PC synapses under *in vitro* conditions involves presynaptic CB1 receptors (Llano et al., 1991a; Yoshida et al., 2002), NMDA receptors (Duguid and Smart, 2004), and postsynaptic GABA_A_ receptors (Hirano and Kawaguchi, 2014). In the present study, MLI–PC GABAergic transmission LTD was induced by 1 Hz, but not 2 Hz or 4 Hz, sensory stimulation *in vivo* in mice, suggesting that sensory stimulation-evoked LTD of MLI–PC synaptic transmission is stimulation-frequency-dependent. The cerebellar MLI network acts as a low-pass filter during the processing of high-frequency sensory information, and plays a critical role in the sensory-related outputs of PCs in the cerebellar cortex (Chu et al., 2012; Bing et al., 2015a). However, cerebellar PCs generate sensory-related output that is limited to low-frequency sensory stimulation, and is independent of GABA_A_ receptor-mediated inhibition (Bing et al., 2015b). Therefore, MLI–PC GABAergic transmission LTD was induced by 1 Hz, but not 2 Hz or 4 Hz, sensory stimulation.

In terms of cerebellar cortical circuitry, MLIs receive sensory information from the MF–GC–PF pathway, which evokes strong GABAergic inhibition rather than excitation in cerebellar PCs (Chu et al., 2011a,b). Therefore, LTD of MLI–PC GABAergic transmission was induced by depression of the sensory stimulation-evoked spike firing of MLIs, such as the sensory stimulation-evoked LTD of MF–GC synapses and/or PF–MLI synapses. Indeed, it is reported that sensory stimulation at 4 Hz can induce LTD at MF–GC synapses in anesthetized rats (Roggeri et al., 2008). PF–PC LTD could occur together with PF–MLI LTP and MLI–PC inhibitory LTP, while PF–PC LTP could occur together with PF–MLI LTD and MLI–PC inhibitory LTD (Gao et al., 2012). However, our results show that air-puff stimulation at 1 Hz does not change the properties of sensory-evoked spike events on MLIs, suggesting that GABAergic transmission LTD is induced by repeated sensory stimulation at MLI–PC synapses.

### Possible Mechanisms of Sensory-Induced MLI–PC GABAergic LTD *In Vivo* in Mice

Our present results show that MLI–PC GABAergic LTD can be prevented by blocking CB1 receptor activity, and can be pharmacologically induced by a CB1 receptor agonist, indicating that sensory stimulation induces GABAergic transmission LTD at MLI–PC synapses via the eCB signaling pathway. eCB-mediated LTD, has been reported to be induced in several brain areas at both inhibitory and excitatory synapses (Chevaleyre et al., 2006). In the hippocampus, stimulation of Schaffer collaterals induces CB1 receptor-dependent LTD at presynaptic GABAergic terminals via activation of mGluR1 (Chevaleyre et al., 2007). In the cerebellar cortex, eCB release from other PCs can be triggered by activation of mGluR1 following the spontaneous CF inputs (Safo et al., 2006). In the cerebellar cortex, CB1 receptor-dependent PF–PC presynaptic LTD was observed *in vivo* in the absence of a pharmacological blocker, suggesting that eCB signaling under *in vivo* conditions is stronger than that under *in vitro*. A presynaptically expressed form of eCB-dependent LTD has been suggested at PF–MLI synapses (Soler-Llavina and Sabatini, 2006). Therefore, eCB signaling plays a critical role in cerebellar cortical neuronal plasticity.

Additionally, mGluR1 activation could induce eCB generation and release from cerebellar PCs and MLIs (Brown et al., 2003; Beierlein and Regehr, 2006; Soler-Llavina and Sabatini, 2006). However, our present results show that MLI–PC GABAergic transmission LTD can be induced in the presence of an mGluR1 blocker, indicating that mGluR1 plays a non-essential role during facial stimulation-evoked LTD of MLI–PC GABAergic transmission of PCs. In fact, facial stimulation evokes inhibition rather than excitation in cerebellar PCs under control conditions (Chu et al., 2011a,b); thus, the inhibition of PCs during sensory stimulation resulted in a lower amount of eCB released by activation of mGluR1. Interestingly, blockade of NMDA receptors during 1 Hz facial stimulation abolished the expression of MLI–PC GABAergic LTD, indicating that the sensory stimulation induced eCB-dependent LTD of GABAergic transmission at MLI–PC synapses via activation of NMDA receptors. Although electrophysiological recordings have shown that functional NMDA receptors are no longer expressed in cerebellar PCs after the first postnatal week (Konnerth et al., 1990; Llano et al., 1991b), postsynaptic functional NMDA receptors are prominent in CF–PC synapses in adult mice (Piochon et al., 2007; Renzi et al., 2007; Bidoret et al., 2009). Importantly, NMDA receptor subunits have been found in cultured MLIs (Duguid and Smart, 2004) and on the axonal pinceau of basket-type MLIs (Petralia et al., 1994). The presence of NMDA receptors on MLIs is the likely a source of eCB (Beierlein and Regehr, 2006) for presynaptic LTD (Qiu and Knöpfel, 2009; Li and Burrell, 2011). Indeed, NMDA receptor activation is required for eCB-dependent presynaptic LTD induction when presynaptic LTP is pharmacologically blocked both in mouse cerebellar slices (Qiu and Knöpfel, 2009) and *in vivo* in mice (Chu et al., 2014). The present results show that the expression of MLI–PC GABAergic LTD is dependent on the activity of NMDA receptors, but that 1 Hz sensory stimulation did not induce a significant change in the properties of sensory-evoked spike events on MLIs, suggesting that LTD of GABAergic transmission might involve presynaptic NMDA receptors. Presynaptic NMDA receptors are thought to act as local high-gain glutamate detectors in cerebellar MLIs (Rossi and Collin, 2013), and the activation of presynaptic NMDA receptors in MLIs can induce an increase in GABA release into MLI–PC terminals (Bouhours et al., 2011) Additionally, activated NMDA receptors trigger the activation of nitric oxide (NO) synthesis and the release of NO from MLIs (Akazawa et al., 1994; Carter and Regehr, 2000). NO works as retrograde signal, and has been implicated in various forms of presynaptically expressed LTP (Hardingham and Fox, 2006; Qiu and Knöpfel, 2007; Chu et al., 2014). However, it seems that NO contributes less to the LTD of GABAergic transmission at MLI–PC synapses under *in vivo* conditions (data not shown).

Taken together, our present study demonstrates that 1 Hz, but not 2 Hz or 4 Hz, facial stimulation induces an eCB-mediated LTD of GABAergic transmission at MLI–PC synapses via activation of NMDA receptors in cerebellar cortical Crus II *in vivo* in mice. Our results highlight that eCB retrograde signaling is activated by sensory stimulation and is necessary for the induction of LTD of MLI–PC GABAergic transmission in living animals.

## Conflict of Interest Statement

The authors declare that the research was conducted in the absence of any commercial or financial relationships that could be construed as a potential conflict of interest.
